# Formulation Development and Evaluation of Pravastatin-Loaded Nanogel for Hyperlipidemia Management

**DOI:** 10.3390/gels8020081

**Published:** 2022-01-28

**Authors:** Gaurav Kant Saraogi, Siddharth Tholiya, Yachana Mishra, Vijay Mishra, Aqel Albutti, Pallavi Nayak, Murtaza M. Tambuwala

**Affiliations:** 1 Sri Aurobindo Institute of Pharmacy, Indore 453111, Madhya Pradesh, India; gauravsaraogi13@gmail.com; 2 SVKM’s NMIMS School of Pharmacy & Technology Management, Shirpur 425405, Maharashtra, India; siddhart619@gmail.com; 3 Department of Zoology, Shri Shakti Degree College, Sankhahari, Ghatampur, Kanpur Nagar 209206, Uttar Pradesh, India; yachanamishra@gmail.com; 4 School of Pharmaceutical Sciences, Lovely Professional University, Phagwara 144411, Punjab, India; pallavinayak97@gmail.com; 5 Department of Medical Biotechnology, College of Applied Medical Sciences, Qassim University, Buraydah 52571, Saudi Arabia; 6 School of Pharmacy and Pharmaceutical Sciences, Ulster University, Coleraine, Londonderry BT52 1SA, UK; m.tambuwala@ulster.ac.uk

**Keywords:** nanogel, hyperlipidemia, pravastatin, polymer

## Abstract

Hyperlipidemia is a crucial risk factor for the initiation and progression of atherosclerosis, ultimately leading to cardiovascular disease. The nanogel-based nanoplatform has emerged as an extremely promising drug delivery technology. Pravastatin Sodium (PS) is a cholesterol-lowering drug used to treat hyperlipidemia. This study aimed to fabricate Pravastatin-loaded nanogel for evaluation of its effect in hyperlipidemia treatment. Pravastatin-loaded chitosan nanoparticles (PS-CS-NPs) were prepared by the ionic gelation method; then, these prepared NPs were converted to nanogel by adding a specified amount of 5% poloxamer solution. Various parameters, including drug entrapment efficacy, in vitro drug release, and hemolytic activity of the developed and optimized formulation, were evaluated. The in vitro drug release of the nanogel formulation revealed the sustained release (59.63% in 24 h) of the drug. The drug excipients compatibility studies revealed no interaction between the drug and the screened excipients. Higher drug entrapment efficacy was observed. The hemolytic activity showed lesser toxicity in nanoformulation than the pure drug solution. These findings support the prospective use of orally administered pravastatin-loaded nanogel as an effective and safe nano delivery system in hyperlipidemia treatment.

## 1. Introduction

The term ‘nanogel’ is defined as the nanosized particles formed by a cross-linked polymer physically or chemically. It was first introduced by cross-linked bifunctional networks of a polyion and a non-ionic polymer for the delivery of polynucleotides [1]. Nanogels are typical formulations with a size range of 20–200 nm [2]. The volume fraction can be altered to maintain the three-dimensional (3D) structure by varying solvent quality and branching. Since gene delivery has become possible within cellular organelles for gene silencing therapy, nanogels have transformed the field of gene therapy [1]. As a result of their size, they can evade renal clearance and have a longer serum half-life. Nanogels tend to absorb a lot of water or physiological fluid without affecting their internal network structure. For targeted drug delivery, stimulus-responsive drug release, or the development of composite materials, chemical changes can be made to integrate a large number of ligands [3]. Nanogels have several characteristics that contribute to their effectiveness as a delivery system. They have exceptional thermodynamic stability, high solubilization capacity, low viscosity, and the ability to withstand rigorous sterilization techniques [4,5]. The majority of nanogel systems are made up of cross-linked synthetic polymers or natural biopolymers. Small molecules or biomacromolecules can be incorporated into the pores of the 3D network in nanogels. Polymeric nanogels as drug carriers have the advantages of artificially controlling drug dosage via external stimuli, concealing disagreeable odor of drug, enhancing therapeutic efficacy, and decreasing the drug’s side effects. For example, anticancer drugs and proteins are excellent for administration via chemically cross-linked or physically constructed nanogel systems because they have severe side effects, a short circulation half-time, and are easily degradable by enzymes [6,7,8].

Hyperlipidemia is described as an abnormally high amount of lipids and lipoproteins in the blood, and it is thought to be a major risk factor for accelerated atherosclerosis and, as a result, cardiovascular disease. In hyperlipidemia, cholesterol levels, particularly low-density lipoprotein cholesterol (LDL-C), are often increased and serve as the primary therapeutic target [9].

As the result of a lack of LDL receptors in hepatocytes, hyperlipidemia, a disease linked to atherosclerosis, can develop [10,11]. Since injected polymeric NPs are quickly taken up by hepatic Kupffer cells, LDL-absorbing NPs may have improved LDL transport to the liver. At the same time, intrinsic toxicity can also be treated with NPs. These NPs have unique physicochemical properties, which have shown promising drug delivery systems (DDS) to the desired sites in the body. These enhance the drug release, improve the bioavailability and solubility, and minimize toxicity and drug degradation. Due to the expanded contact region for van der Waals attraction, NPs show strong adhesion. It is necessary to comprehend the pharmaceutically relevant properties of NPs to achieve the better development of the novel DDS [12,13,14]. The major challenge for developing NP as a DDS is controlling the particle size, surface properties, and time-release of the active moiety to get the site-specific action at the desired proportion and dose. Polymeric NPs offer distinct advantages over other nanocarriers; they increase the drugs/protein stability and show beneficial controlled release properties [15,16,17,18,19,20,21,22].

Pravastatin is a cholesterol-lowering agent that belongs to the statin class of drugs. It came from the microbial transformation of mevastatin, which was the first statin ever found. It is a ring-opened dihydroxyacid with a 6′-hydroxyl group that does not need to be activated in the body. When compared to lovastatin and simvastatin, the greater hydrophilicity of pravastatin is expected to give benefits such as reduced penetration across lipophilic membranes of peripheral cells, increased selectivity for hepatic tissues, and a reduction in adverse effects [23]. Pravastatin is structurally identical to 3-hydroxy-3-methylglutaryl (HMG), an endogenous substitute for HMG-coenzyme A reductase (HMG-CoA reductase). Pravastatin, unlike its parent chemical, mevastatin, and statins such as lovastatin and simvastatin, does not require in vivo activation. Its hydrolyzed lactone ring resembles the tetrahedral intermediate of reductase, allowing it to bind with far more affinity than its normal substrate. The bicyclic component of pravastatin binds to the coenzyme A portion of the active site. Pravastatin works in two ways to reduce cholesterol levels. First, it causes modest decreases in intracellular cholesterol pools due to its reversible suppression of HMG-CoA reductase activity. The number of LDL receptors on the cell surfaces increases, resulting in improved receptor-mediated degradation and clearance of circulating LDL. Second, pravastatin inhibits LDL production by inhibiting the hepatic synthesis of VLDL, the LDL precursor [24,25,26,27].

## 2. Results and Discussion

### 2.1. Experimental Design for Optimization of Nanoparticles

The 3^2^ level central composite design (CCD) with results is shown in Table 1 and Table 2. All the batches were formulated and evaluated for particle size and entrapment efficiency. The obtained results provided considerable useful information and confirmed the utility of the statistical design for the conduction of the experiments. Independent variables such as polymer amount (mg), the stirring speed, and probe sonication time significantly influenced the observed responses, particle size (nm), and entrapment efficiency (%). The optimized formulation batch was determined by systematic analysis of data using design expert software.

Pravastatin-loaded CSNPs (PS-CS-NPs) were prepared by an ionic gelation method using a probe sonicator. Pravastatin is a BCS class-III drug having high solubility and low permeability. The influence of the amount of chitosan, stirring speed, and probe sonication time at different concentrations and time, respectively, was investigated in the ionic gelation method with a fixed quantity of tripolyphosphate (TPP) and amount of drug. In the prescreening study, chitosan and poloxamer were selected as polymer and stabilizer/gelling agents, respectively. This study found that chitosan and poloxamer gave a desired particle size for all the batches, which was found to be 400–3155 nm with a lower polydispersity index (PDI). The zeta potential ensures the stability of the formulation. The stirring speed was taken with the help of the literature search. The dependent variable, i.e., entrapment efficiency obtained at various levels of three independent variables (A, B, C), was observed. The polynomial equation was obtained in terms of actual factors for entrapment efficiency (%). The correlation coefficient (R^2^) value of the polynomial equation was found to be 0.9566, indicating a good fit (Table 2).

**Figure 1:**
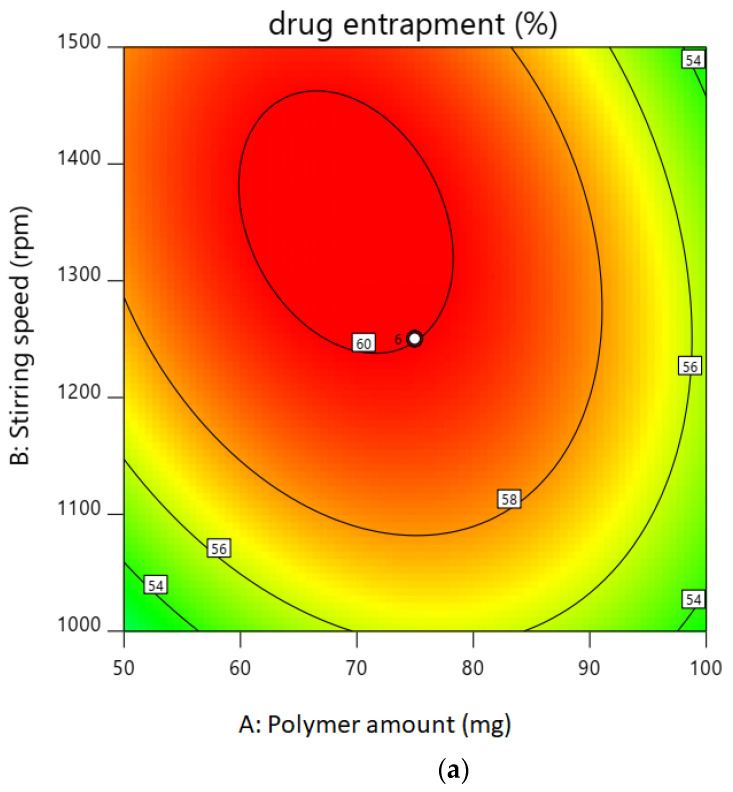

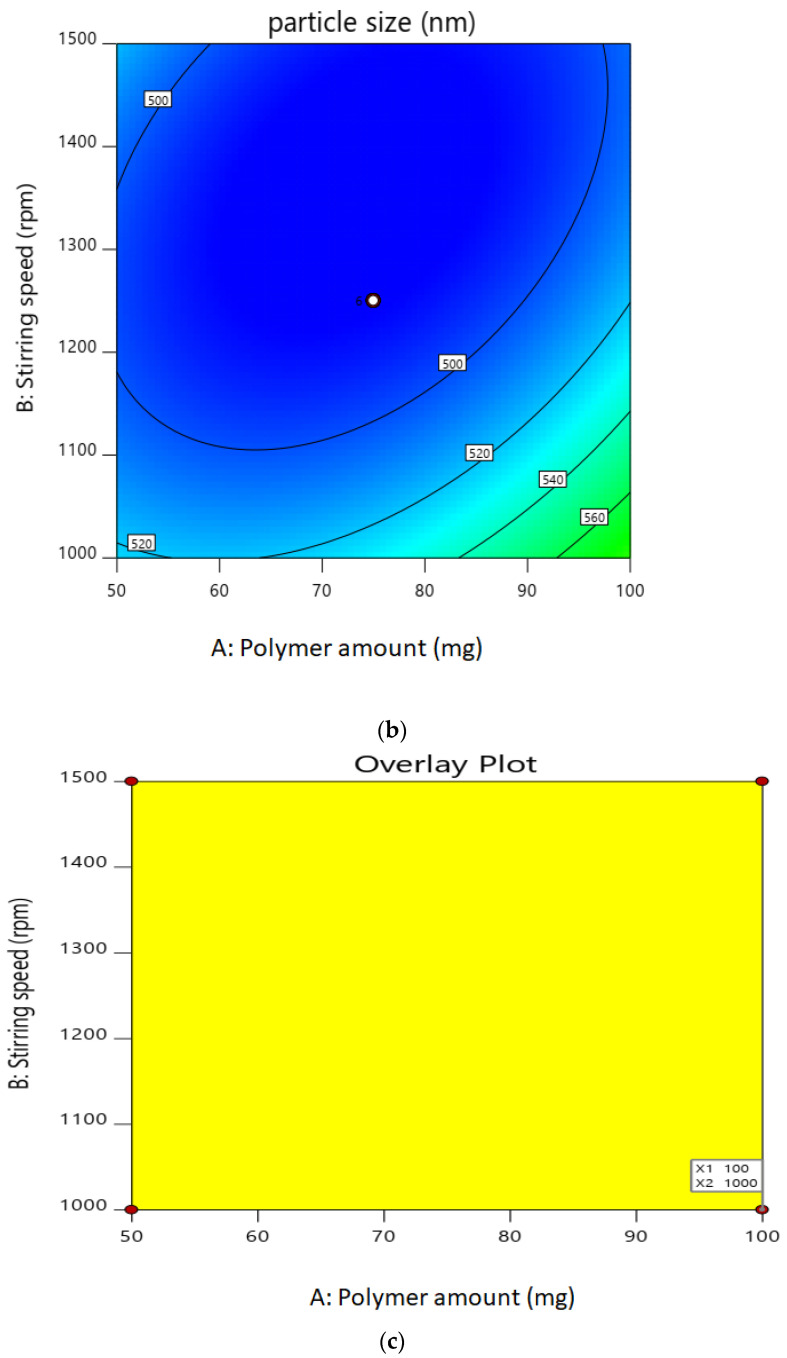
(**a**). Contour plot of drug entrapment. (**b**). Contour plot of particle size. (**c**). Overlay plot for confirmation.

The entrapment efficiency for all the batches was calculated, and the wide variation was observed, i.e., the values ranged from 47 to 60%. Hence, the obtained results indicate that the entrapment efficiency value was strongly affected by the variables used in this study. It can be found that A, B, (A^2^), and (C^2^) are the significant model, as the *p* values of the above independent variable are less than 0.05. Here, the coefficient of independent variable B, (A^2^), and (C^2^) has the negative value. An increase in stirring speed or average stirring speed and probe sonication time increases the entrapment efficiency as it increases the viscosity, which leads to a decrease in the particle size of the nanoparticles. As the viscosity of the external aqueous phase decreases, effective diffusion of the organic phase is hindered, leading to smaller droplet formed, which affects the mean size of the particle, and hence, the entrapment efficiency is high. This result is also confirmed by the contour plot for the entrapment efficiency in Figure 1a,b. The desirability of optimized batch is shown in Figure 1c overlay plot.

### 2.2. Characterization of Optimized Nanoparticles

#### 2.2.1. Particle Size

**Figure 2:**
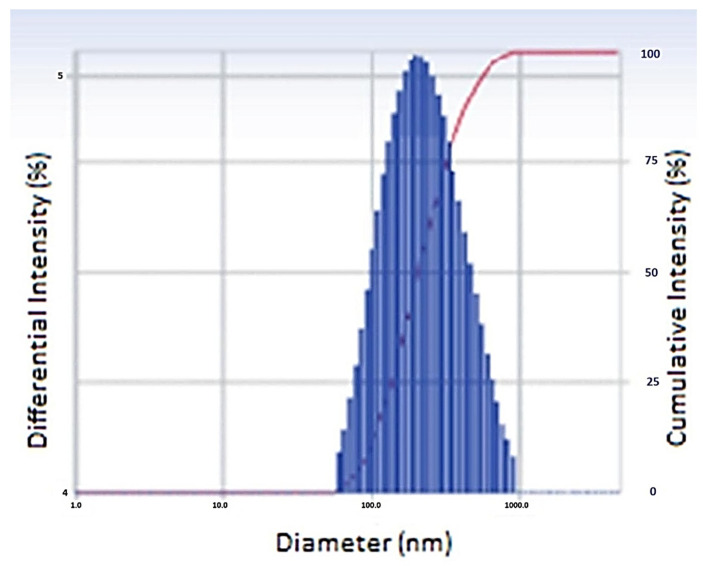
Particle size of optimized nanoparticles.

The optimized batch (N20) had a Z-average particle size of 486 nm and PDI of 0.303, indicating that the NPs were distributed uniformly. Figure 2 showed the particle size distribution pattern of the optimized NPs formulation.

#### 2.2.2. Entrapment Efficiency

The entrapment efficiency of the optimized batch was found to be 50%. Hence, the results indicate that the measured value obtained for entrapment efficiency was as expected. It is confirmed that at a 95% level of confidence, the values obtained for the optimized batch was in range. The predicted values for entrapment efficiency and particle size are shown in Table 3.

#### 2.2.3. Scanning Electron Microscopy (SEM) of Nanoparticles

**Figure 3:**
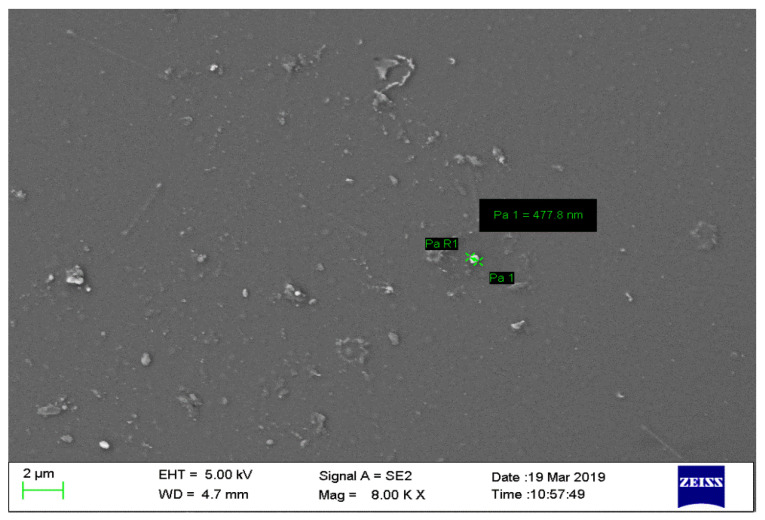
Scanning Electron Microscopy image of chitosan nanoparticles showing regular shape.

The SEM image showed that the chitosan nanoparticles have a regular shape (Figure 3).

#### 2.2.4. In Vitro Drug Release

**Figure 4:**
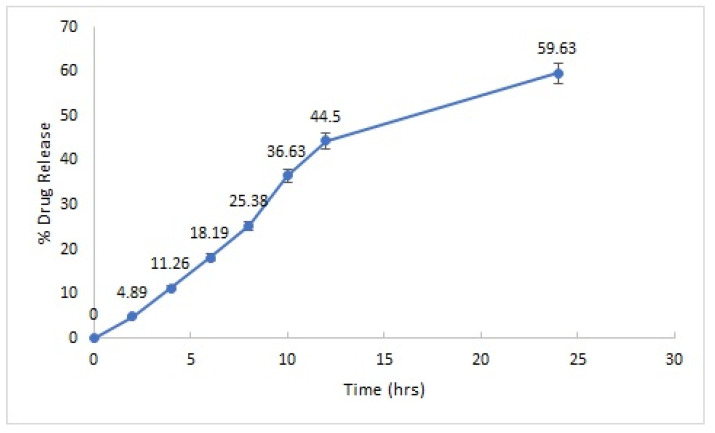
In vitro drug release profile of optimized PS-CS-NG formulation.

The optimized PS-CS-NG formulation was subjected for in vitro drug release behavior. It was performed by the dialysis bag method, and the optimized formulation (N20) showed the highest drug release (59.63%) in 24 h (Figure 4). This showed the uniform distribution of drug in the formulation.

#### 2.2.5. Swelling Ratio

At pH 6.8 and 7.4, the swelling ratio for nanogel preparation was determined to be 259% and 382%, respectively. The high swelling ratio showed that the nanogels had a good capacity for accessing and keeping water molecules within the polymer network, and it was clear that this capacity was lower in pH 6.8 buffers than in pH 7.4 buffers. The hydrophilic nature of CS and the nature of bonds inside the matrix structure determine the equilibrium water content of nanogels. Furthermore, the hydrogen bonds between water molecules and functional groups present in nanogel structures, such as hydroxyl groups, ether, amine, and unhydrolyzed acetamide linkages, are the most important controlling factors during swelling. Since the functional groups or matrix structure of CS would alter significantly across the two buffers, a lower swelling ratio in pH 6.8 buffer was most likely attributable to weaker hydrogen bonding than in pH 7.4 buffer. These findings imply that the pH of CS-based nanogels is a significant factor in their swelling and that a high pH is desired.

#### 2.2.6. SEM Image of Structural Network of Nanogel

**Figure 5:**
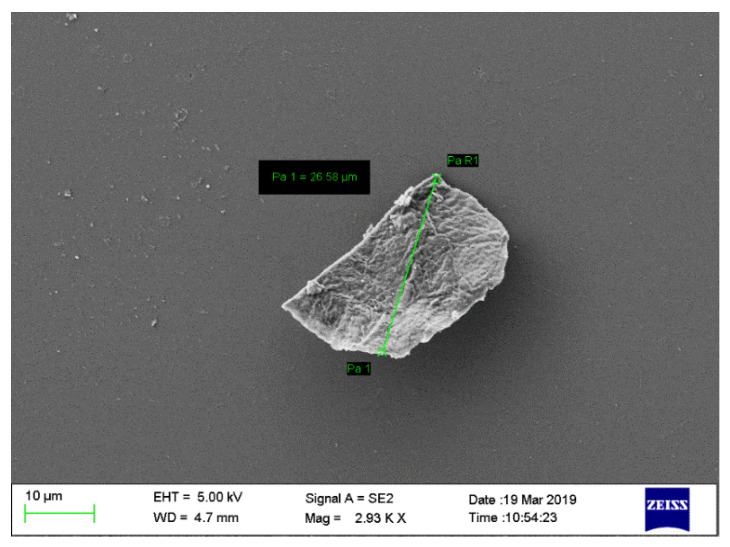
Scanning electron microscopy image of structural network of nanogel revealing a regular and uniform quasi-shape of NPs.

As a result of the uneven structure of chitosan, SEM image revealed that the NPs have a regular and uniform quasi-shape in the structural network of nanogel (Figure 5).

#### 2.2.7. Effect on Surface Morphology of Erythrocytes

**Figure 6:**
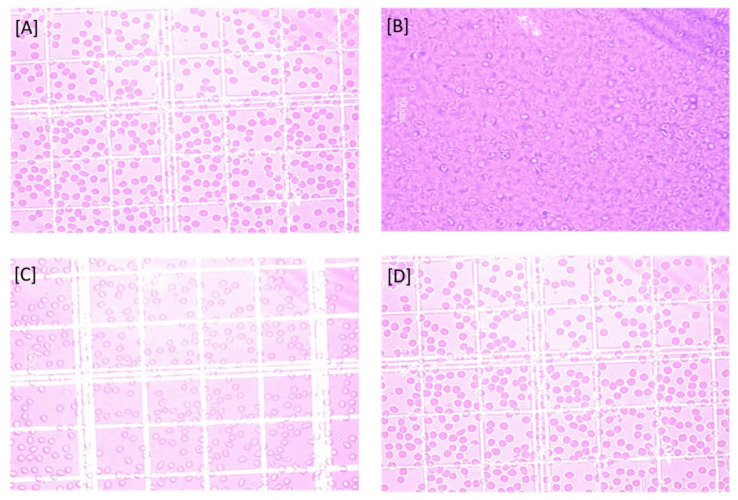
Erythrocytes on interaction with (**A**) Saline solution, (**B**) Water, (**C**) Pure drug solution, and (**D**) Nanogel formulation.

On interaction with aqueous solution of plain drug as compared to normal saline as well as nanogel formulation, the erythrocytes showed the changes in shape and their surface morphology, as depicted in Figure 6. Based on the effects on erythrocytes’ surface morphology and hemolytic toxicity investigations, it can be concluded that PS-CS-NGs demonstrated promising performance and can be developed as a carrier system for further biomedical application in other routes of drug delivery.

#### 2.2.8. Hemolytic Toxicity

The percentage hemolytic toxicity level of the samples was found to be within the range. The drug-containing nanogel (PS-CS-NG) formulation was found to be less toxic (6.08%) than the pure drug solution (7.2%).

#### 2.2.9. Pharmacokinetic Studies

The plasma concentration–time profile obtained from a single oral administration of pravastatin oral solution and pravastatin oral nanogel to Swiss albino rats was used to calculate the pharmacokinetic parameters (Table 4). According to these findings, the optimized pravastatin oral nanogel formulation has higher bioavailability than the pravastatin oral solution.

Some of the major observations and conclusion that could be drawn with the experimental work are represented in Table 5.

## 3. Conclusions

The main aim of the present investigation was to develop and optimize the novel pravastatin nanogel using central composite design. The optimized batch showed the desired characteristics and in vitro drug release of the nanogel formulation showing the sustained release. Forced Degradation Studies (FDS) have also indicated about the stability of drug in oxidative and neutral medium (Figures S1 and S2). Pravastatin belongs to cholesterol inhibitor antihyperlipedemia. It inhibits the cholesterol absorption and acts on the brush border of the small intestine. As the drug belongs to BCS class III, inadequate permeability has always been a difficult hurdle to overcome, as it is a critical aspect in a drug’s bioavailability after oral administration. Since a rising number of newly produced Statin series medication candidates in pre-clinical development belong to BCS class III, formulation options to circumvent this issue are in high demand. Over the last decade, drug formulation as NPs/nanogel has received a lot of attention as one of the numerous strategies to improve a product’s permeability/dissolution rate features with the goal of increasing its oral bioavailability. The hypothesis underlying dissolution rate improvement, when considering drug particle size reduction to the nanometer range, is that the resulting drug-loaded nanogel has a substantially larger effective surface area. Ionic gelation is one of the easiest and most effective strategies for reducing drug particle size to the nanoscale range among the different available technologies.

## 4. Materials and Methods

### 4.1. Materials

Gift sample of Pravastatin Sodium drug was received from Biocon Pvt. Ltd., Bengaluru, India. Chitosan was purchased from HiMedia Pvt. Ltd., Mumbai, India. Glacial acetic acid was purchased from Merck, Mumbai, India. Pentasodium tripolyphosphate and Poloxamer were purchased from Sigma Aldrich, Mumbai, India. All other used chemicals and reagents were of analytical grade.

### 4.2. Methods

#### 4.2.1. Preparation of Pravastatin-Loaded Chitosan Nanogel

Pravastatin-loaded chitosan nanoparticles (PS-CS-NPs) were prepared by the ionic gelation method [28]. Briefly, chitosan was dissolved in 10 mL of 0.5% *v*/*v* acetic acid with constant stirring. Tripolyphosphate (TPP) or sodium triphosphate and pravastatin dissolved in 10 mL of deionized water was added drop wise under constant magnetic stirring. The sample was kept for constant magnetic stirring for 2 h. The obtained solution was turbid, and the NPs were obtained. An appropriate amount of poloxamer (5% solution) was added in above prepared NPs and kept for stirring for about half an hour. This led to the formation of nanogel [29].

#### 4.2.2. Experimental Design for Optimization of Nanogel

Pravastatin-loaded chitosan nanogels (PS-CS-NGs) were prepared by an ionic gelation method using the quality by design (QbD) approach [28,29]. For obtaining the desired product quality, critical quality attributes (CQA) were identified from the quality target product profile (QTPP). The CQAs of the PS-CS-NGs are listed in Table 6. During the QbD-based development of PS-CS-NGs, an initial risk assessment (RA) effort was also carried out, which involved evaluation of those formulation variables that could have an impact on CQAs. Table 7 shows the level of initial risk assessment for individual formulation variables.

Particle size and its uniform distribution are the ultimate benchmark for the novel DDS. On the basis of the preliminary trials and risk assessment, a 3^2^ level CCD was employed to study the effect of Polymer amount (X1), Stirring speed (X2), and Sonication time (X3) as independent variables (Table 8) while Particle size (Y1) and Entrapment efficiency (Y2) were the dependent variables. Nanogel was prepared as per the experiment design matrix generated by the software (Design-Expert^®^ Software Version 11).

#### 4.2.3. Characterization and Evaluation of Optimized Nanoparticles/Nanogel

Nanoparticles are characterized based on their size, morphological characteristics, and surface charge. The size distribution, average particle diameter, and surface charge influence the physical stability, redispersibility, and in vivo performance of NPs.

##### Particle Size and Zeta Potential

The particle size distribution and morphology are the primary determinants of NP characterization. Particle size was determined at 25 °C by the photon correlation spectroscopy technique [30]. This analysis measures the particle size of particles suspended in liquids in the range of 0.6 nm to 10 µm with sample suspension concentrations from 0.00001 to 40%. All the data presented are produced under identical production conditions. The interaction of NPs with the biological environment, as well as their electrostatic interaction with bioactive chemicals, is determined by their surface charge and intensity. To maintain particle stability and avoid aggregation, zeta potential levels (high zeta potential values, either positive or negative) are attained.

##### Entrapment Efficiency

The obtained formulation was dissolved in methanol and centrifuged in cooling microfuge at 20 °C for 15 min. After centrifugation, the supernatant was collected and filtered. Then, 0.2 mL of this stock solution was diluted with water up to 1 mL. The un-entrapped pravastatin was estimated and analyzed by the UV method (Perkin Elmer series 200) at 238 nm. Drug entrapped was estimated by subtracting un-entrapped drug from the total drug. Entrapment efficiency was further calculated from the entrapped drug.

##### Morphology Observation

With direct observation of the NPs, the electron microscopy-based approach evaluates their size, shape, and surface morphology. The solution of NPs was first converted into a dry powder, which was then deposited on a sample holder before being sputter-coated with a conducting metal (such as gold). The entire sample was scanned with a focused fine beam of electrons for analysis, and surface characteristics were determined.

##### Fourier-Transform Infrared Spectroscopy

FT-IR spectroscopy was used to investigate drug–polymer interactions. FT-IR studies were performed on both the pure drug and the excipients (Figures S3 and S4). Physical mixes were also scanned in the wavelength range of 400–4000 cm^−1^ in an FT-IR spectrophotometer, and the spectra were recorded.

##### In Vitro Drug Release

The most adaptable and widely used approach for determining drug release from nanosized dosage forms is dialysis. The dialysis bag was filled with 10 mL of optimized nanogel formulation and was immersed immediately in the 50 mL of phosphate buffer saline (PBS) pH 7.4. The sampling for drug release was performed at definite time intervals. The sample (5 mL) was withdrawn at each time interval, and the withdrawn volume was replaced by fresh PBS to maintain sink condition. The samples were analyzed UV spectrophotometrically.

##### Swelling Ratio

The swelling ratio is a measurement of the capability of a nanogel to absorb water. All samples reached the equilibrium water content within 1 h, and the swelling ratio values were determined at pH 6.8 and pH 7.4 [31].

##### Hemolytic Toxicity

Hemolytic toxicity was determined using the previously described method [32,33]. In a nutshell, whole human blood was collected in a HiAnticlot blood collection vial from a healthy donor. The blood was washed with PBS pH 7.4, which was followed by separation of the erythrocytes by centrifugation at 3000 rpm for 5 min, whereby the supernatant was pipette off repeatedly (*n* = 3), and the erythrocytes were suspended in normal saline solution to obtain 10% hematocrit. Then, 1 mL of RBC suspension was incubated with distilled water (taken as 100% hemolytic standard) and normal saline (taken as blank spectrophotometric estimation). In this study, Afterwards, 1 mL of erythrocytes suspension and 1 mL of formulation were taken in separate tubes and the volume made up to 10 mL with normal saline. Similarly, 1 mL of drug solution was mixed with 9 mL of normal saline and interacted with erythrocytes suspension. The tubes were allowed to stand for 1 h at 37 °C with intermittent shaking. The tubes were centrifuged for 15 min at 3000 rpm, and the absorbance of supernatants was measured at 308 nm, which was used to estimate the percentage of hemolysis using 100% hemolytic standard obtained with distilled water diluted; similarly, the percent hemolysis was calculated for each sample by using the following Equation (1):

Hemolysis = ABs/AB_100_ × 100 (1)

##### Effect on Surface Morphology of Erythrocytes

Erythrocyte suspension was treated with drug and nanogel formulation at a specified concentration (1 mg/mL), and the morphological status of erythrocytes was assessed under an optical microscope (Leica, DMLB, Heerbrugg, Switzerland) [32,33].

##### Pharmacokinetics Studies

After a one-week acclimatization period, albino rats weighing 120 ± 10 g were employed in the experiment. All animal experiments were carried out in accordance with the protocol approved by Institutional Animal Ethics Committee (IAEC) of NMIMS School of Pharmacy & Technology Management, Shirpur (Maharashtra), India (SPTM-IAEC/Dec-18/03/03; dated 16 December 2018). A pharmacokinetics investigation was carried out using an optimized nanogel formulation and an in-house made immediate release solution. Each rat received a dose of pravastatin sodium corresponding to 10 mg per kg of body weight. Oral solution was made by dissolving an equivalent amount of drug in water in order to provide the drug dose based on the animal’s body weight. The animals were placed into two groups, each with three animals. An i.m. injection of a 1:5 mixture of xylazine (1.9 mg/kg) and ketamine (9.3 mg/kg) was used to lightly anaesthetize the animals. A solution providing the required dose based on the rat’s body weight was given orally to one group. Another group received an optimized nanogel with the required dose based on the rat’s body weight. A 26-gauge needle was used to take 1 mL of blood from the marginal ear vein every 1 h for up to 10 h. To separate plasma, blood samples were centrifuged at 8000 rpm for 10 min at 15 °C (REMI Pvt. Ltd., Vasai, India). Until further examination, plasma samples were kept at −20 °C. The plasma samples were analyzed for the pravastatin using HPLC method (Figure S5).

## Figures and Tables

**Table 1:** Design table for optimized batch formulation.

		Factor 1	Factor 2	Factor 3	Response 1	Response 2
Std	Run	A: Polymer Amount	B: Stirring Speed	C: Sonication Time	Drug Entrapment	Particle Size
		(mg)	(rpm)	(min)	%	(nm)
1	10	50	1000	5	53.6	556
2	11	100	1000	5	56	602
3	17	50	1500	5	58.95	604
4	16	100	1500	5	47	614
5	15	50	1000	10	49.5	556
6	5	100	1000	10	47	655
7	7	50	1500	10	55	500
8	6	100	1500	10	54.6	498
9	14	33	1250	7.5	51	543
10	13	117	1250	7.5	50.33	543
11	20	75	830	7.5	50.68	578
12	1	75	1670	7.5	56.95	502
13	4	75	1250	3	58.2	566
14	12	75	1250	12	54	601
15	8	75	1250	7.5	60	486
16	18	75	1250	7.5	60	486
17	3	75	1250	7.5	60	486
18	9	75	1250	7.5	60	486
19	2	75	1250	7.5	60	486
20	19	75	1250	7.5	60	486

**Table 2:** Analysis of variance (ANOVA) for factorial model at *p* < 0.05 level of significance.

Source	Sum of Squares	df	Mean Square	F-Value	*p*-Value	
Model	356.02	9	39.56	9.40	0.0008	Significant
A—Polymer amount	13.50	1	13.50	3.21	0.1035	
B—Stirring speed	29.27	1	29.27	6.96	0.0248	
C—Sonication time	19.97	1	19.97	4.75	0.0544	
AB	18.76	1	18.76	4.46	0.0609	
AC	5.53	1	5.53	1.31	0.2784	
BC	35.07	1	35.07	8.34	0.0162	
A^2^	165.68	1	165.68	39.38	<0.0001	
B^2^	74.72	1	74.72	17.76	0.0018	
C^2^	31.10	1	31.10	7.39	0.0216	
Residual	42.07	10	4.21			
Lack of Fit	42.07	5	8.41			
Pure Error	0.0000	5	0.0000			
Cor Total	398.09	19				

**Table 3:** Confirmation table of optimized nanoparticles.

Response	Predicted Mean	Predicted Median	Std Deviation	N	SE Prediction	95% Low	Mean	95% High
Entrapment efficiency	49.4784	49.4784	2.05155	1	2.65049	49.368	49.4784	55.384
Particle size	647.994	647.994	23.6573	1	30.5699	579.88	647.994	716.108

**Table 4:** Summary of values of peak plasma concentration (C_max_), time of C_max_ attainment (t_max_), Area under curve (AUC), and Mean residence time (MRT) of pravastatin oral solution and pravastatin oral nanogel.

S. No.	Time (h)	Pravastatin Oral Solution (µg/mL)	Pravastatin Oral Nanogel (µg/mL)
1	0	0	0
2	1	68	14
3	2	47	26
4	3	23	39
5	4	11	79
6	5	0	65
7	6		47
8	7		31
9	8		9
10	10		0
	C_max_	68 µg/mL	79 µg/mL
	t_max_	1 h	4 h
	AUC last	140.707 (µg·h/mL)	302.022 (µg·h/mL)
	AUC total	155.85 (µg·h/mL)	312.912 (µg·h/mL)
	T _half_	0.98 h	0.83 h
	MRT	2.35 h	4.62 h

**Table 5:** Concluding features of experimental work for optimized formulation.

Parameters	Result	Inference
Particle size	486.2 nm	Desired and acceptable size
Polydispersity index	0.303	Uniform distribution
Zeta potential	43.4 mV	Positively and evenly distributed
Entrapment efficiency	50%	Nanoparticles leads for higher entrapment of drug
Drug release	59.63% (24 h)	Sustained release of the drug was obtained
SEM studies	-	Regular shape
Compatibility study	Characteristic peak is obtained	Overlay plot confirms the characteristic peaks of drug
Toxicity study	Less toxicity is exhibited	Safer to use
Pharmacokinetic study	Higher bioavailability	

**Table 6:** Critical Quality Attributes (CQA) parameters.

CQAs	Polymer Amount	Stirring Speed	Sonication Time
Particle size	Low	Medium	High
% Entrapment efficiency	Low	Medium	High

**Table 7:** Quality by Design (QbD) details.

Profile	Target	Justification
Dosage form	Nanogel	Novel dosage form for targeted drug delivery
Dosage design	Sustained release oral nanogel	For increasing residence time of pravastatin
Therapeutic indication	Antihyperlipidemia	Pravastatin acts by inhibition of cholesterol producing enzymes
Route of administration	Oral	Most suitable route of administration and can be well absorbed in intestine
Particle size	10–1000 nm	Drug absorption and uniform biodistribution
Zeta potential	−200 to 200 mV	Needed to ensure stability
Entrapment efficiency	>50%	Nanogel entraps higher amount of drug

**Table 8:** Independent variables for Quality by Design (QbD).

Independent Variables	Low	High
Coded Values	(−1)	(+1)
A = Polymer amount (mg)	50	100
B = Stirring speed (rpm)	1000	1500
C = Sonication time (min)	4	8

## Data Availability

Data are contained within the article or supplementary material.

## Supplementary Materials

The following supporting information can be downloaded at: https://www.mdpi.com/article/10.3390/gels8020081/s1, Figure S1: Hydrolytic degradation chromatogram of pravastatin sodium, Figure S2: Oxidative degradation chromatogram of pravastatin sodium, Figure S3: FTIR Spectrum of pravastatin sodium, Figure S4: Characteristics peaks of compatibility study, Figure S5: HPLC chromatogram of pravastatin sodium.
