# Supplementary material for: Formulation Development and Evaluation of Pravastatin-Loaded Nanogel for Hyperlipidemia Management
Source: Gels. 2022 Jan 28;8(2):81. doi: 10.3390/gels8020081 (PMC8871575; doi:10.3390/gels8020081)
Supplement: Supplementary file 1 [file gels-08-00081-s001.zip › gels-1425947-supplementary.pdf]

Supplementary information

1. **Forced Degradation Studies (FDS)**

Chemical stability of pharmaceutical molecules is a matter of great concern as it affects the safety and efficacy of the drug product. The FDA and ICH guidelines state the requirement of stability testing data to understand how the quality of a drug substance and drug product changes with time under the influence of various environmental factors. Knowledge of the stability of molecule helps in selecting proper formulation and package as well as providing proper storage conditions and shelf-life, which is essential for regulatory documentation. Forced degradation is a process that involves degradation of drug products and drug substances at conditions more severe than accelerated conditions and thus generates degradation products that can be studied to determine the stability of the molecule. The ICH guidelines state that stress testing is intended to identify the likely degradation products which further helps in determination of the intrinsic stability of the molecule and establishing degradation pathways, and to validate the stability indicating procedures used.

But these guidelines are very general in conduct of forced degradation and do not provide details about the practical approach towards stress testing. Although forced degradation studies are a regulatory requirement and scientific necessity during drug development, it is not considered as a requirement for formal stability program. It has become mandatory to perform stability studies of new drug moiety before filing in registration dossier. The stability studies include long term studies (12 months) and accelerated stability studies (6 months). But intermediate studies (6 months) can be performed at conditions milder than that used in accelerated studies. So the study of degradation products like separation, identification and quantitation would take even more time. As compared to stability studies, forced degradation studies help in generating degradants in much shorter span of time, mostly a few weeks. The samples generated from forced degradation can be used to develop the stability indicating

method which can be applied latter for the analysis of samples generated from accelerated and long term stability studies.

1.1 Hydrolytic degradation of drug Pravastatin sodium

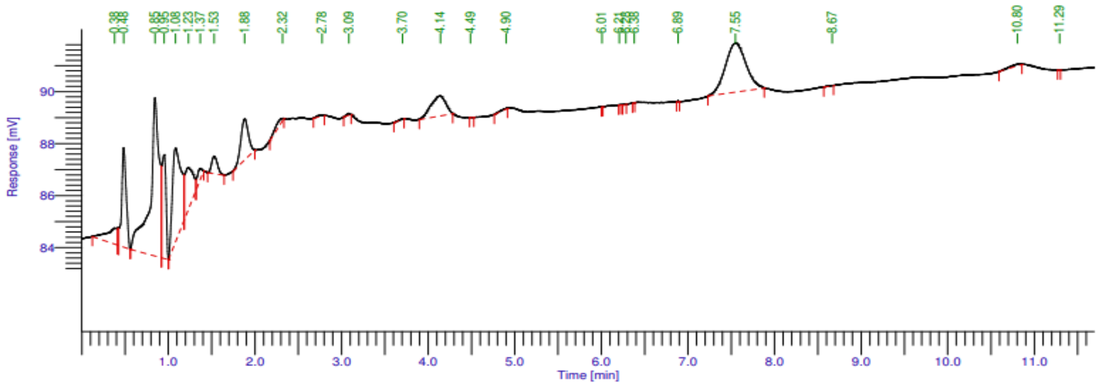

Figure S1 Hydrolytic Degradation Chromatogram of Pravastatin sodium

1.2 **Oxidative degradation of drug Pravastatin sodium**

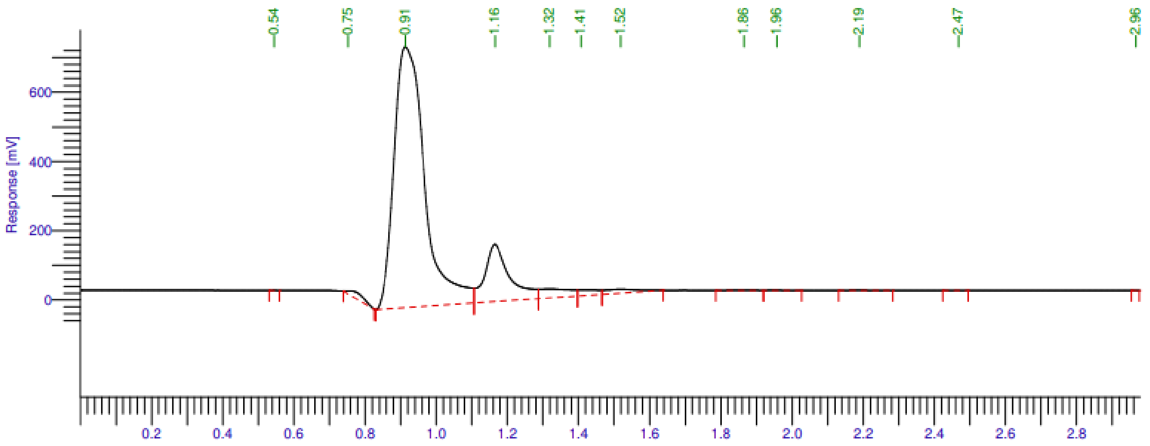

Figure S2 Oxidative Degradation Chromatogram of Pravastatin sodium

Degradation results

**Conditions**	**% Degradation**
Hydrolytic	94%
Oxidative	26.3%

The forced degradation study indicated that the drug is easily degradable at or into acidic and basic conditions while it is stable in oxidative conditions.

2. Identification test

An FTIR spectrum was scanned for Pravastatin Sodium in the IR range from 400 – 4000 cm^{-1}. The characteristic peaks were obtained as given in the Figure S1.

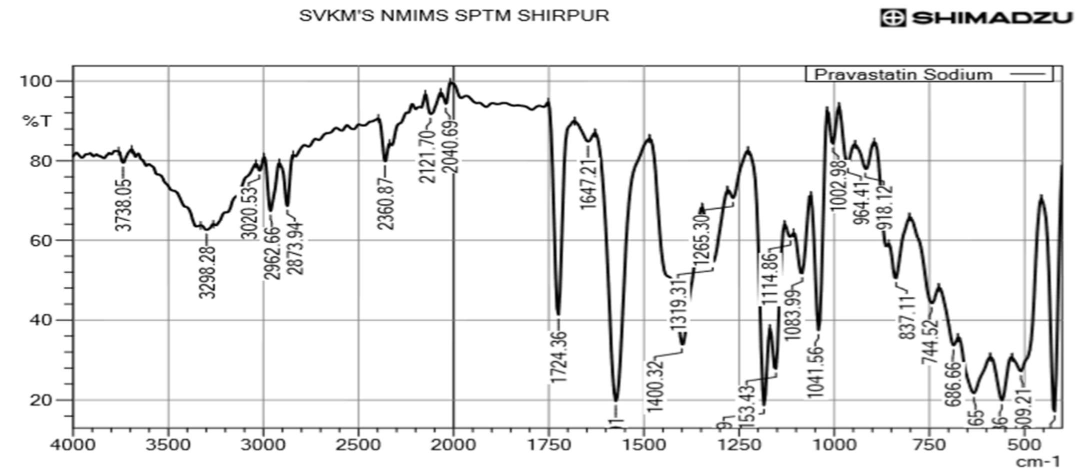

Figure S3. FTIR Spectrum of Pravastatin Sodium

Functional group	Wave number (cm^{-1})
O-H bond of Carboxylic group	3650-2700
Strong Aliphatic Group	3000-2850
C=O Bond	1760-1690
C=C Bond	1600-1500

3. Compatibility Study

FTIR analysis was recorded to study the chemical reaction between Pravastatin, Chitosan, TPP and Poloxomer FTIR spectra were scanned for Pravastatin and another for the physical mixture of Pravastatin, Chitosan, TPP and Poloxomer (formulation) in the IR range from 400 – 4000 cm^{-1}. FTIR spectra were recorded at room temperature to

verify alteration in frequency and intensity of bands of pure drug in the presence of excipients (Figure S2).

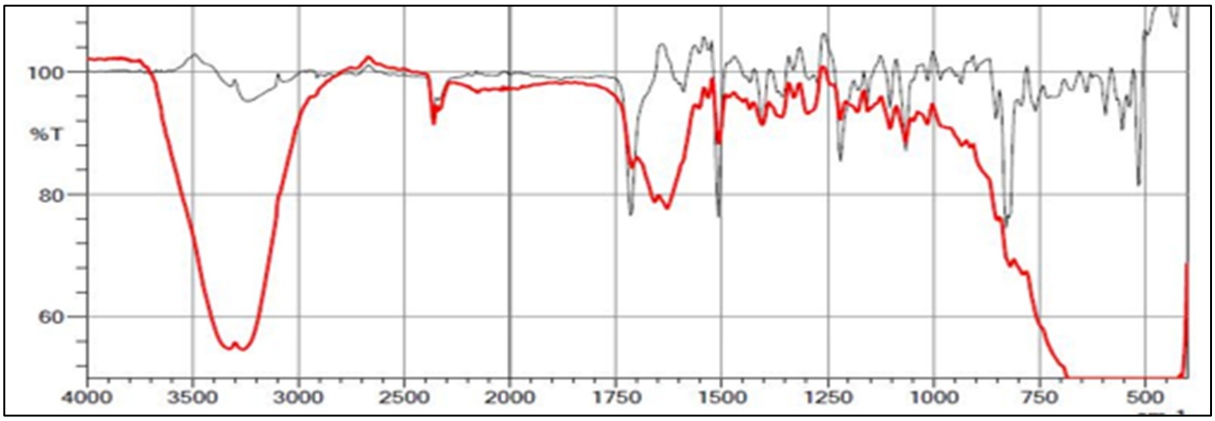

Figure S4. Characteristics peaks of compatibility study

Functional Group	Wave number (cm^{-1})
O-H carboxylic acid group	3650-2700
Strong aliphatic group	3000-2850
C=O bond	1760-1690
C=C bond	1600-1500
p- di substituted benzene	1000-800

4. HPLC chromatogram of Pravastatin Sodium

The retention time of the drug was found to be 1.19 minutes

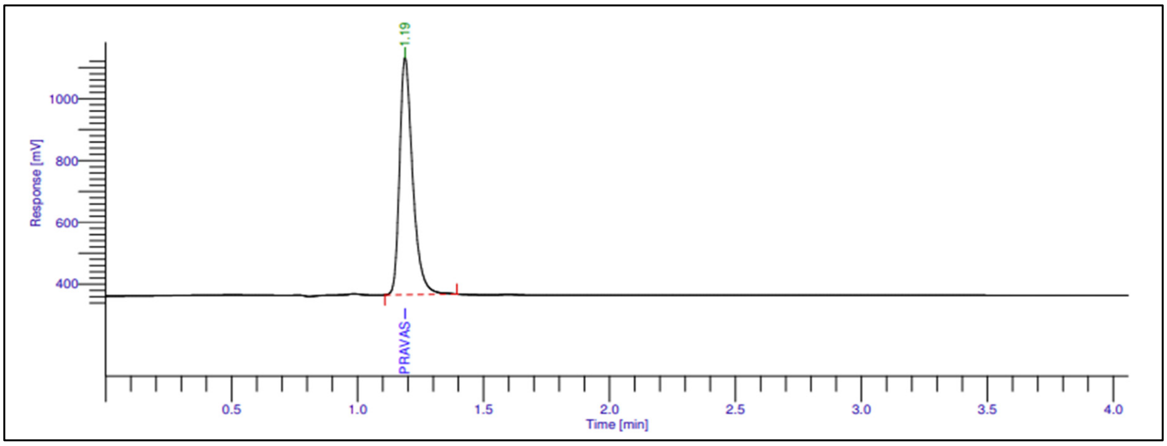

Figure S5. HPLC chromatogram of Pravastatin Sodium
